# Inhalable TPGS/DPPC Micelles Coloaded with Curcumin and Icariin for Targeted Lung Cancer Therapy

**DOI:** 10.1021/acsomega.5c00008

**Published:** 2025-04-11

**Authors:** Chengwei Jiang, Rongjun Bai, Satyanarayana Somavarapu

**Affiliations:** Department of Pharmaceutics, School of Pharmacy, University College London, 29-39 Brunswick Square, London WC1N 1AX, U.K.

## Abstract

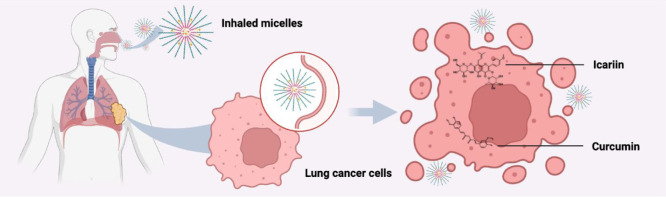

Lung cancer, particularly NSCLC, poses a major therapeutic challenge due to drug resistance and the poor aqueous solubility of chemotherapeutic agents, limiting treatment efficacy. This study investigates inhalable micelles for the codelivery of curcumin (CUR) and icariin (ICA), two hydrophobic bioactive compounds with anticancer potential, as a targeted therapeutic approach for NSCLC. The optimized micellar formulation (9:1 TPGS/DPPC) yielded nanomicelles (∼18 nm) with high encapsulation efficiency (∼90%) and a zeta potential of −1.24 mV, demonstrating stability for pulmonary administration. *In vitro* cytotoxicity studies demonstrated enhanced anticancer activity of CUR- and ICA-loaded micelles against A549 lung cancer cells (IC_50_ = 3.0 μg/mL), lower than doxorubicin (30 μg/mL), suggesting enhanced cytotoxic potential. Additionally, DPPH assays confirmed that encapsulation preserved curcumin’s functionality. Aerosolization studies demonstrated a high fine particle fraction (67 ± 3%) and emitted fraction (95 ± 1.0%), confirming the micelles’ suitability for deep lung deposition and effective pulmonary drug delivery. These findings highlight the potential of CUR- and ICA-loaded micelles as an inhalable NSCLC treatment, requiring further preclinical investigation.

## 1 Introduction

Lung cancer remains the leading cause of cancer-related mortality, with over 1.8 million projected deaths worldwide in 2021.^1^ Nonsmall cell lung cancer (NSCLC) accounts for approximately 80%–85% of all cases.^2^ Standard treatment modalities for NSCLC include surgery, radiotherapy, chemotherapy, immunotherapy, and molecularly targeted therapy.^3^ The development of biomarker-driven therapies in the past decade has facilitated personalized treatment strategies for NSCLC.^4^ However, despite these advancements, fewer than 25% of patients experience durable therapeutic responses, with resistance often emerging due to tumor heterogeneity, adaptive mutations, and drug efflux mechanisms.^5^ Developing localized drug delivery systems that enable efficient lung deposition and enhance drug retention in the respiratory tract remains essential for NSCLC therapy.

Natural bioactive compounds, including phytochemicals, have been explored for their potential role in cancer therapy due to their ability to interact with tumor microenvironments and support conventional treatments.^6−8^ Building upon these encouraging outcomes, we aim to explore novel therapeutic approaches for NSCLC utilizing natural products.

Curcumin (CUR), a polyphenolic bioactive compound extracted from *Curcuma longa*, has demonstrated anticancer activity across multiple malignancies, including colorectal, pancreatic, breast, prostate, lung, and oral cancers.^9^ Its pharmacological effects are primarily attributed to its ability to regulate apoptosis, cell cycle progression, and inflammatory signaling pathways.^10^ Curcumin has demonstrated synergistic effects when combined with chemotherapeutic agents such as gemcitabine, platinum-based agents, and dasatinib, further enhancing its therapeutic potential.^11−13^

Icariin (ICA), a bioactive flavonoid glycoside isolated from *Epimedium* species, is widely used in traditional Chinese medicine.^14^ Due to its hydrophobic nature, ICA is highly soluble in organic solvents but demonstrates limited aqueous solubility, restricting its direct clinical application.^15,16^ It exhibits anticancer properties by modulating multiple pathways, including the induction of apoptosis, inhibition of cell cycle progression, and suppression of angiogenesis and metastasis.^17^ Moreover, our previous findings indicate that ICA-loaded micelles can regulate macrophage polarization toward the M2 phenotype, a mechanism that holds significant implications for the tumor microenvironment and immune modulation in cancer therapy.^18,19^

Combination therapy involving multiple bioactive agents has been demonstrated to enhance therapeutic efficacy while reducing the risk of drug resistance in oncology.^20,21^ The coadministration of CUR and ICA presents a promising strategy for NSCLC treatment by utilizing their complementary mechanisms of action, including apoptosis induction, cell cycle arrest, and modulation of oncogenic signaling pathways.

The clinical application of CUR and ICA is limited by their poor aqueous solubility, which hinders their formulation into inhalable systems and requires appropriate delivery strategies to improve dispersibility. Nanoparticle-based drug delivery platforms provide an effective approach to overcoming these limitations by improving solubility, prolonging systemic circulation, and enabling site-specific drug accumulation.^22−30^ Various nanoparticle platforms including liposomes,^22^ nanoemulsions,^23^ solid lipid nanoparticles,^24^ metal nanoparticles,^25^ protein nanoparticles,^26^ nanotubes,^27^ nanofibers,^28^ carbon dots,^29^ and polymeric nanoparticle^30^ are being explored for their ability to enhance solubility and provide controlled drug release. These systems can improve drug accumulation in tumor tissues while minimizing systemic side effects, making them promising candidates for the combined delivery of CUR and ICA in NSCLC therapy.

Polymeric micelles are nanosized core–shell structures formed through the self-assembly of amphiphilic macromolecules, such as block and graft copolymers. These micelles consist of a hydrophobic core and a hydrophilic shell, enabling the encapsulation of hydrophobic drugs within the core while the hydrophilic shell provides stability in aqueous environments. Measuring between 10 and 100 nm in size, polymeric micelles offer distinct advantages for drug delivery, including enhanced dispersibility, biocompatibility, increased solubility of water-insoluble drugs, and improved absorption by reducing degradation rates.^31^

D-α-tocopheryl polyethylene glycol succinate (TPGS)-based micellar formulations have emerged as a potential nanomedicine platform for drug delivery. TPGS is a pharmaceutical excipient composed of a hydrophobic vitamin E moiety and a hydrophilic polyethylene glycol (PEG) chain. This amphiphilic structure enhances the solubility of poorly water-soluble compounds such as CUR and ICA. Literature reports have documented TPGS-based micellar formulations loaded with anticancer agents like docetaxel,^32^ paclitaxel conjugated with transferrin,^33^ and formulations targeting the HER-2 receptor for drug delivery,^34^ demonstrating the versatility of TPGS in nanomedicine.

Pulmonary drug delivery is a noninvasive approach that enables localized drug deposition while reducing systemic exposure. This strategy is particularly relevant for inhalable formulations of hydrophobic drugs like CUR and ICA, which require solubilization for effective nebulization.^35^ Dipalmitoylphosphatidylcholine (DPPC), a major component of pulmonary surfactant, has been approved for inhalation in various formulations, including Survanta (as an active ingredient) and Inbrija (as an excipient). The use of DPPC in inhalable formulations enhances lung compatibility and can improve the delivery and absorption of therapeutic agents in the respiratory tract.^35^

Previous studies have demonstrated that CUR and ICA have anticancer activity. However, the synergistic effects of CUR and ICA in cancer therapy, particularly when utilizing nanoparticle-based pulmonary delivery systems, have not been thoroughly investigated. This study aims to develop and characterize CUR and ICA-loaded TPGS/DPPC micelles for pulmonary administration, evaluating their physicochemical properties, aerosol performance, and *in vitro* effects. The addition of DPPC, which resembles natural pulmonary surfactants, may enhance drug absorption in the respiratory tract, thereby optimizing therapeutic outcomes.^36^ This study hypothesizes that CUR and ICA-loaded TPGS/DPPC micelles can be formulated for nebulizer-mediated pulmonary administration, facilitating lung deposition, improving cellular uptake, and enabling sustained therapeutic action against NSCLC.

## 2 Materials and Methods

### 2.1 Materials

Curcumin (Alfa Aesar Co., Ltd., UK), icariin (Tokyo Chemical Industry Co., Ltd., Japan), 2,2-diphenyl-1-picrylhydrazyl (DPPH) (Alfa Aesar Co., Ltd., Japan), coumarin 6 (Sigma-Aldrich, USA), doxorubicin (DOX) (Sigma-Aldrich, USA), methanol (Sigma-Aldrich, USA), d-α-tocopheryl polyethylene glycol succinate (TPGS) (Sigma-Aldrich, USA), dipalmitoylphosphatidylcholine (DPPC) (Lipoid Co., Ltd., Germany), A549 cell lines (ATCC, USA), methanol, DMEM/F12, fetal bovine serum (FBS), PBS, TryLE Express Enzyme (1×), and MTT reagent were purchased from Merck Life Science UK Ltd. (UK). NucBlue Reagent (Hoechst 33342) was obtained from Life Technologies Limited (UK). CellMask Orange Actin Tracking Stain was purchased from Thermo Fisher Scientific (UK). Acetonitrile, HPLC-grade water, and dimethyl sulfoxide (DMSO) were purchased from Cambridge Bioscience (UK).

### 2.2 Preparation of Micelles

**Figure 1:**
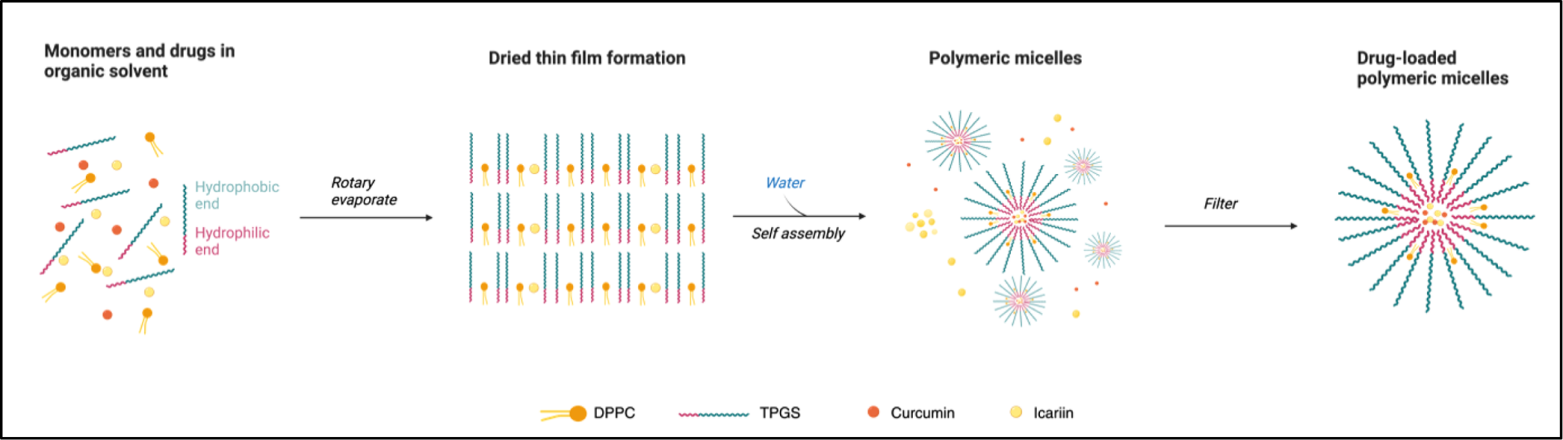
Thin film method for the preparation of ICA and CUR loaded micelles (created in BioRender. Jiang, C. (2025) https://BioRender.com/o68u335).

CUR and ICA loaded micelles were prepared using the thin film hydration method with CUR, ICA, TPGS and DPPC. As shown in Figure 1, 10 mg CUR, 10 mg ICA, and 100 mg of a TPGS/DPPC mixture at different weight ratios (10:0, 9:1, 8:2, and 7:3 w/w) were dissolved with 10 mL of methanol. The solution was transferred to a round-bottom flask and sonicated. The solvent was then removed using a rotary evaporator maintained at 85 °C under reduced pressure to form a thin film on the flask walls. The lipid film was hydrated with purified water and ultrasonicated for 5 min to obtain the micelle formulation. The resulting formulation was filtered through a 0.45 μm filter to remove any unencapsulated drug. An identical preparation method was employed to produce coumarin-6 loaded TPGS/DPPC micelles for the cellular uptake studies.

### 2.3 Particle Diameter, Polydispersity (PDI), and Zeta Potential

The mean particle diameter, polydispersity index (PDI), and zeta potential of the nanoparticles suspended in distilled water were measured using dynamic light scattering (DLS) on a Zetasizer Ultra (Malvern Panalytical Instruments, Malvern, UK). Each sample was introduced into a capillary cell for analysis.

### 2.4 Transmission Electron Microscopy (TEM) Observation

The morphology of the CUR and ICA loaded TPGS/DPPC micelles was examined using transmission electron microscopy (TEM) at an accelerating voltage of 120 kV. Lyophilized micelle samples were reconstituted in distilled water, and a drop of the micellar solution was placed onto a copper grid. The grid was stained with a 2% (w/v) phosphotungstic acid solution, air-dried, and then analyzed by TEM.

### 2.5 Fourier Transform Infrared (FT-IR) Analysis

Fourier Transform Infrared (FT-IR) spectroscopy was employed to analyze CUR, ICA, TPGS/DPPC, and freeze-dried CUR/ICA/TPGS/DPPC powders. Spectra were recorded using a Spectrum 100 FT-IR Spectrometer (PerkinElmer, Waltham, MA, USA). Each sample was scanned in triplicate in transmission mode, collecting both transmittance and absorbance data. The spectral range spanned from 4000 to 650 cm^–1^ with a resolution of 4 cm^–1^, coadding 32 interferograms per measurement. Frequency accuracy was maintained at 0.01 cm^–1^, ensured by the instrument’s internal reference laser.

### 2.6 X-Ray Powder Diffraction (XRD) Analysis

X-Ray Powder Diffraction (XRD) analysis was conducted to investigate the physical state of the CUR and ICA encapsulated micelles, blank micelles, free ICA, and free CUR. Diffractograms were obtained using a MiniFlex600 X-ray diffractometer (Rigaku, Tokyo, Japan) equipped with a copper Kα radiation source. Samples including CUR powder, ICA powder, and freeze-dried powders of blank TPGS/DPPC micelles as well as CUR and ICA-loaded micelles were analyzed in step-scan mode at a current of 15 mA and a voltage of 40 kV. Data were collected over a 2θ range of 3–50°, with a step size of 0.05° and a scan speed of 2° min^–1^.

### 2.7 Differential Scanning Calorimetry (DSC) Analysis

Differential scanning calorimetry (DSC) was utilized to assess the thermal properties of ICA, CUR, freeze-dried blank TPGS/DPPC micelles, and freeze-dried CUR/ICA-loaded micelles. Approximately 5 mg of each sample was placed in an aluminum sample pan and sealed. Thermal analysis was performed using a Q2000 DSC (TA Instruments, New Castle, DE, USA) under a nitrogen atmosphere. The temperature was increased from 25 to 300 °C at a heating rate of 10 °C min^–1^.

### 2.8 Analysis of Molecular Interactions Using ^1^H NMR Spectroscopy

Proton nuclear magnetic resonance (^1^H NMR) spectroscopy was performed to investigate potential molecular interactions between CUR, ICA, and the TPGS/DPPC micelle components. Freeze-dried samples of drug-loaded micelles, a physical mixture of CUR and ICA, and freeze-dried blank micelles were dissolved in deuterated methanol (CD_3_OD). ^1^H NMR spectra were recorded using a Bruker Avance III NMR spectrometer operating at 400 MHz to detect any chemical shifts or spectral changes indicative of interactions

### 2.9 Determination of Encapsulation Efficiency and Drug Loading

Encapsulation efficiency (EE) and drug loading (DL) were determined by analyzing the concentrations of CUR and ICA in methanol-diluted CUR + ICA/TPGS/DPPC micelle samples using high-performance liquid chromatography (HPLC). HPLC-UV (Agilent Technologies 1200 series, USA) equipped with a reversed phase column Zorbax Eclipse XDB-C18 4.6 × 50 mm (Agilent, UK). The mobile phase consisted of 50% HPLC grade water (Sigma-Aldrich, UK) and 50% acetonitrile (Sigma-Aldrich, UK). The injection volume and flow rate were set up at 10 μL and 1 mL/min, and the signal was detected at 270 nm wavelength. The HPLC chromatograms of ICA and CUR combination (Figure S1), calibration curve of ICA concentration (Figure S2), and calibration curve of CUR concentration (Figure S3) have been shown in Supporting Information.

The encapsulation efficiency (EE) was calculated using the following formula:
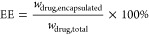
1where *w*_drug,encapsulated_ is the weight of encapsulated drug and *w*_drug,total_ is the total weight of drug added.

The drug loading (DL) was quantified by measuring the drug quantity per 1 mg of freeze-dried formulation:
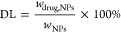
2where *w*_drug,NPs_ is the weight of drug in the nanoparticles and *w*_NPs_ is the weight of the nanoparticles.

### 2.10 *In Vitro* Aerosolization of CUR + ICA/TPGS/DPPC Micelles

The lung deposition pattern of the CUR and ICA loaded TPGS/DPPC micelles was evaluated using a Next Generation Impactor (NGI) (MSP Corporation, USA). The components and stages of the NGI were thoroughly cleaned with methanol and allowed to air-dry naturally. In accordance with the European Pharmacopoeia guidelines (Chapter 2.9.44), the NGI, including its collection stages and induction port, was refrigerated at 5 °C for 90 min prior to use.

The airflow rate was maintained at 15L/min, and a filter was inserted into the micro-orifice collector (MOC). The flow rate was verified using a flow meter (DFM2000, Copley Scientific Limited, Nottingham, UK) before initiating the experiments. For each test, 2 mL of freshly prepared CUR + ICA/TPGS/DPPC micelle suspension was loaded into the reservoir of a nebulizer connected to the induction port of the NGI. The nebulizer was activated, and aerosolization continued until the formulation was completely nebulized.

The amount of CUR + ICA/TPGS/DPPC deposited on each of the seven NGI collection cups was quantitatively extracted by rinsing with 5 mL of methanol. Deposits in the induction port and residual formulation inside the nebulizer chamber were also collected by rinsing with an appropriate volume of methanol. The concentrations of CUR and ICA on each stage were quantified using high-performance liquid chromatography (HPLC), and aerosolization parameters were calculated accordingly.

The emitted fraction (EF) represents the percentage of the total mass of CUR emitted from the nebulizer after each impactor run:
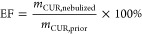
3where *m*_CUR,nebulized_ is the mass of nebulized CUR and *m*_CUR,prior_ is the mass of CUR present in the nebulizer before nebulization.

The fine particle dose (FPD) is defined as the mass of particles with aerodynamic diameters less than 5 μm within the total emitted dose; this corresponds to the formulation deposited on stages 4–7 of the NGI at an airflow rate of 15 L/min, with cutoff diameters ranging from 3.30 to 0.98 μm.^37^

The fine particle fraction (FPF) is defined as the percentage of FPD in comparison to the total recovered dose:^38^
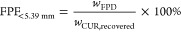
4where *w*_FPD_ is the weight of the FPD and *w*_CUR,recovered_ is the total weight of CUR recovered in all stages.

The mass median aerodynamic diameter (MMAD) is the particle size at which 50% of the cumulative mass of the aerosolized particles is smaller and 50% is larger. This parameter was determined from a plot of the cumulative mass fraction versus the effective cutoff diameter on a logarithmic probability scale. The geometric standard deviation (GSD) quantifies the dispersion of the aerodynamic particle size distribution and was calculated using the same plot employed for MMAD determination, following standard equations:
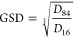
5where *D*_84_ and *D*_16_ describe the diameters of which 84% and 16% of the aerosol mass is included, respectively.

### 2.11 Antioxidant Assay by DPPH

The assay mixture contains 1.5 mL of 0.1 mM DPPH methanolic solution, methanolic solutions of various concentrations of CUR, ICA, CUR + ICA, and CUR + ICA loaded micelles in a total volume of 2 mL. Controls (0.5 mL of methanol and 1.5 mL of DPPH solution) were also taken. The mixture was incubated at 25 °C for 30 min, avoided from light. The reduction of absorbance was measured spectrophotometrically at 516 nm.

The free radical scavenging activity is expressed as IC_50_ values, the concentration of the sample required for 50% of the free radical to be scavenged. It is calculated according to the following equation”
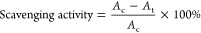
6where *A*_c_ is the absorbance of the control and *A*_t_ is the absorbance of the test solution.

### 2.12 Cell Culture

A549 cell lines were cultivated in DMEM/F12 enriched with 10% fetal bovine serum (FBS) at 37 °C in a humidified environment containing 5% CO_2_. A549 cells were regularly cultured to about 80% confluence in T75 flasks, subculture using 0.25% trypsin (Gibco, UK), and subsequently seeded in 96-well or 6-well plates for further investigations.

### 2.13 Cell Viability by MTT Assay

Cell viability was assessed using the MTT assay. A549 cells were seeded in 96-well plates. Following treatment with ICA, CUR, DOX, blank TPGS/DPPC micelles, and ICA + CUR loaded TPGS/DPPC micelles, 0.5 mg/mL MTT was added to the medium for 4 h at 37 °C. The medium was then removed, and 100 μL DMSO was added to each well to dissolve the formazan crystals. Optical density (OD) was measured at 570 nm using a microplate reader. The survival ratio was expressed as a percentage of the control.

### 2.14 Cellular Uptake Through Confocal Microscope

A549 cells were seeded at a density of 1 × 10^4^ cells/well in 6-well plates and treated with 2 mg/mL coumarin-6 loaded micelles at 37 °C for 2 h. After washing twice with PBS (pH 7.4), cell nuclei were stained with Hoechst dye, and images were captured using a confocal microscope (LSM 710 Confocal Microscope, Thermo Fisher Scientific, UK).

### 2.15 Quantitative Measurement of Cellular Uptake through Flow Cytometry

The cellular uptake of TPGS/DPPC micelles by A549 cells was measured using flow cytometry utilizing coumarin-6 labeling. A549 cells were cultured in 6-well plates and exposed to coumarin-6 loaded nano micelles at a dosage of 0.5 mg/mL at 37 °C for 2 h. The cells were subsequently washed twice with PBS, collected using 0.25% trypsin, spun at 1500 rpm for 5 min, resuspended in PBS, and the mean fluorescence intensity was quantified using a flow cytometer (Attune NxT Acoustic Focusing Cytometer, Thermo Fisher Scientific, UK).

### 2.16 Statistical Analysis

All values were expressed as the mean ± standard division of the mean. Statistical differences were assessed by *t* test on Prism 9 software. *p* < 0.05 denoted as significant difference.

## 3 Results and Discussion

### 3.1 Size, PDI, Zeta Potential, Drug Loading, and Encapsulation Efficiency

CUR + ICA nanoparticles were prepared using the thin-film hydration method, a scalable and reproducible technique known for cost-effectiveness.^39,40^ TPGS, serving as a surfactant, solubilizer, and permeation enhancer, improved drug encapsulation efficiency and solubility of hydrophobic drugs.^40^ DPPC, a major pulmonary surfactant component, is widely used in drug delivery due to its role in lung retention.^35,41^ However, as DPPC primarily forms liposomes rather than micelles, TPGS was selected as the main micelle-forming agent.

As presented in Table 1, all CUR + ICA-loaded nanoparticles exhibited mean sizes below 40 nm. Particle size increased with higher DPPC content, with TPGS/DPPC 10:0 (w/w) yielding the smallest size (12 nm) and 7:3 (w/w) the largest (31 nm). Increasing DPPC content resulted in larger particle sizes, consistent with previous findings attributing this to DPPC’s bilayer-forming properties.^42^ For blank nanoparticles (Blank-NPs), sizes ranged from 12.4 to 34 nm, following a similar trend. Encapsulation of CUR and ICA did not significantly alter particle size, confirming formulation stability.

**Table 1:** Particle Size, PDI Values and Zeta Potential of Different Formulations

	Particle size (nm)	PDI	Zeta potential (mV)
Blank-NPs 10:0	12.41 ± 0.08	0.06 ± 0.02	0.03 ± 0.17
Blank-NPs 9:1	17. 50 ± 0.46	0.21 ± 0.02	-0.15 ± 0.42
Blank-NPs 8:2	24.47 ± 1.03	0.34 ± 0.02	0.04 ± 0.15
Blank-NPs 7:3	33.04 ± 1.40	0.48 ± 0.01	-0.05 ± 0.28
CUR + ICA-NPs 10:0	12.46 ± 0.03	0.20 ± 0.01	0.08 ± 0.14
CUR + ICA-NPs 9:1	15.79 ± 1.70	0.21 ± 0.06	-1.24 ± 1.50
CUR + ICA-NPs 8:2	27.31 ± 0.61	0.30 ± 0.02	-0.51 ± 0.36
CUR + ICA-NPs 7:3	31.04 ± 0.80	0.23 ± 0.01	-1.89 ± 0.67

The PDI remained below 0.31, indicating a uniform particle size distribution, critical for stable pulmonary drug deposition.^43^ A PDI ≤ 0.2 is generally preferred for polymer-based nanoparticles to prevent aggregation and improve stability.^44^ The TPGS/DPPC 9:1 (w/w) formulation had a PDI of 0.2, making it optimal for further study.

Additionally, nanoparticles with TPGS/DPPC ratios of 9:1 (w/w) and 7:3 (w/w) exhibited the highest negative zeta potentials (−1.24 mV and −1.89 mV, respectively), which enhances mucus penetration, facilitating pulmonary drug delivery.^45^

As shown in Table 2, drug loading remained consistent (∼18%) across formulations, demonstrating higher efficiency compared to previous CUR-loaded TPGS micelles, such as PEG–PLA/TPGS (DL% = 14%) and TPGS/F127/P123 (DL% = 9%).^46,47^

**Table 2:** Drug Loading (%), Encapsulation Efficiency (%) of CUR and ICA in TPGS/DPPC Micelles

	DL%	EE% (CUR)	EE% (ICA)
CUR + ICA-NPs 10:0	18 ± 3	93 ± 1	97 ± 2
CUR + ICA-NPs 9:1	17 ± 4	84 ± 3	92 ± 4
CUR + ICA-NPs 8:2	18 ± 1	82 ± 3	92 ± 3
CUR + ICA-NPs 7:3	17 ± 1	75 ± 6	85 ± 8

Encapsulation efficiency (EE%) decreased slightly with higher DPPC content. The TPGS/DPPC 9:1 (w/w) formulation exhibited the highest EE% (CUR: 84 ± 3%, ICA: 92 ± 4%), ensuring minimal drug loss and improved formulation consistency.

### 3.2 Micelle Morphology via TEM Analysis

**Figure 2:**
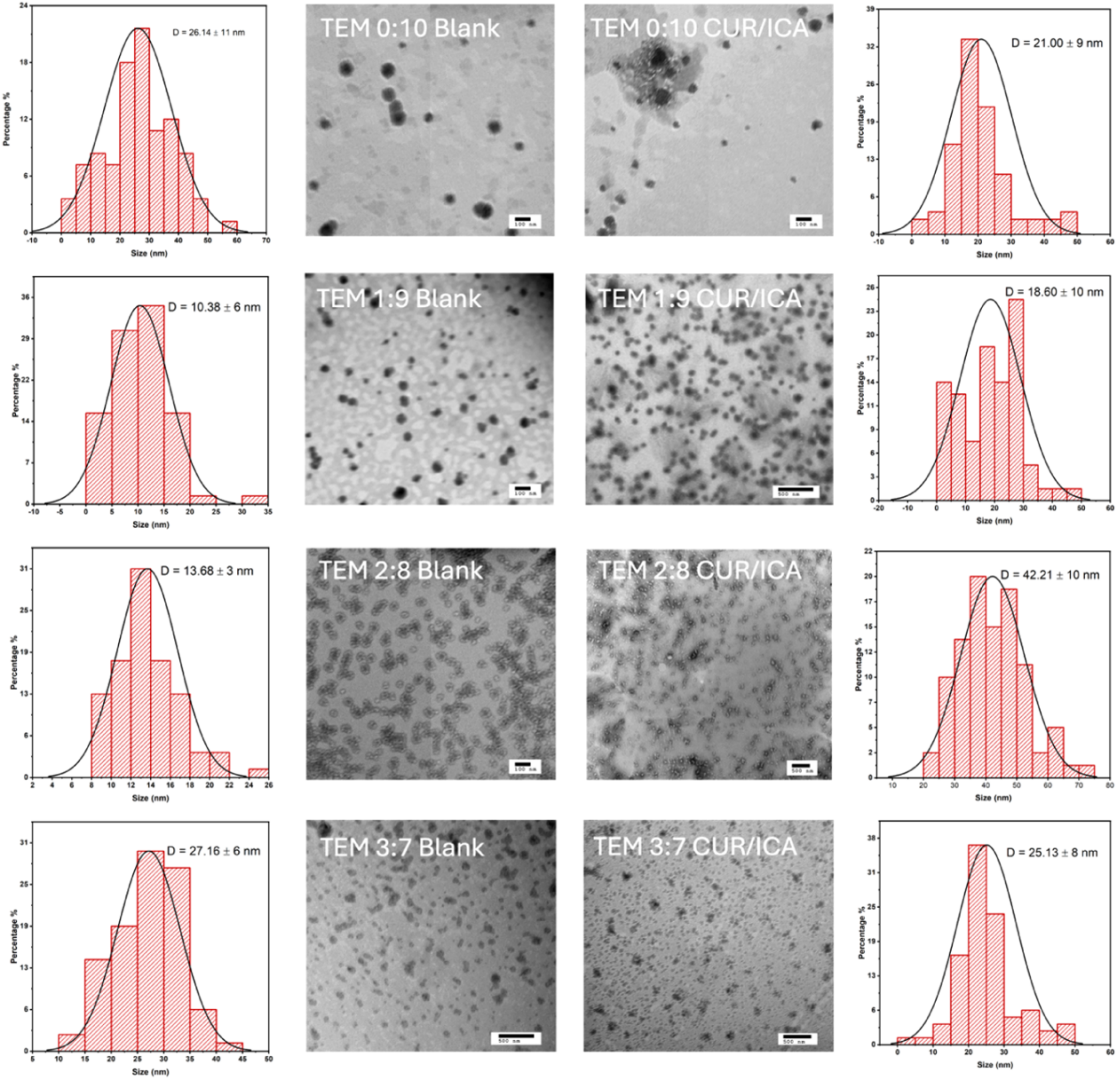
TEM image analysis of blank TPGS/DPPC micelles at different ratios and CUR + ICA-loaded micelles. The figure also presents the particle size distribution curves for both blank and CUR + ICA-loaded TPGS/DPPC micelles, highlighting variations in size across different formulations.

TEM (Figure 2) analysis corroborated DLS measurements, showing micellar structures with comparable size distributions. Across all TPGS/DPPC formulations, no significant deviations in size were observed, with particles ranging between 10 and 40 nm. TEM images displayed spherical micelles with distinct polymeric layers, consistent with micelle formation.

Previous studies indicate that micelles typically exhibit hydrophobic surfactant tails forming a core, while hydrophilic heads face the surrounding aqueous environment, a structural arrangement characteristic of micellar systems within the 10–100 nm size range.^48^ Minimal differences in size between blank and drug-loaded micelles suggest that CUR and ICA encapsulation does not significantly impact micelle structure.

However, nanoparticle aggregation was observed in TPGS/DPPC 8:2 (w/w) and 7:3 (w/w) formulations, correlating with higher PDI values detected in DLS analysis. This aggregation contributes to broader particle size distribution, increasing heterogeneity and PDI values in these formulations.

### 3.3 Stability Study

**Figure 3:**
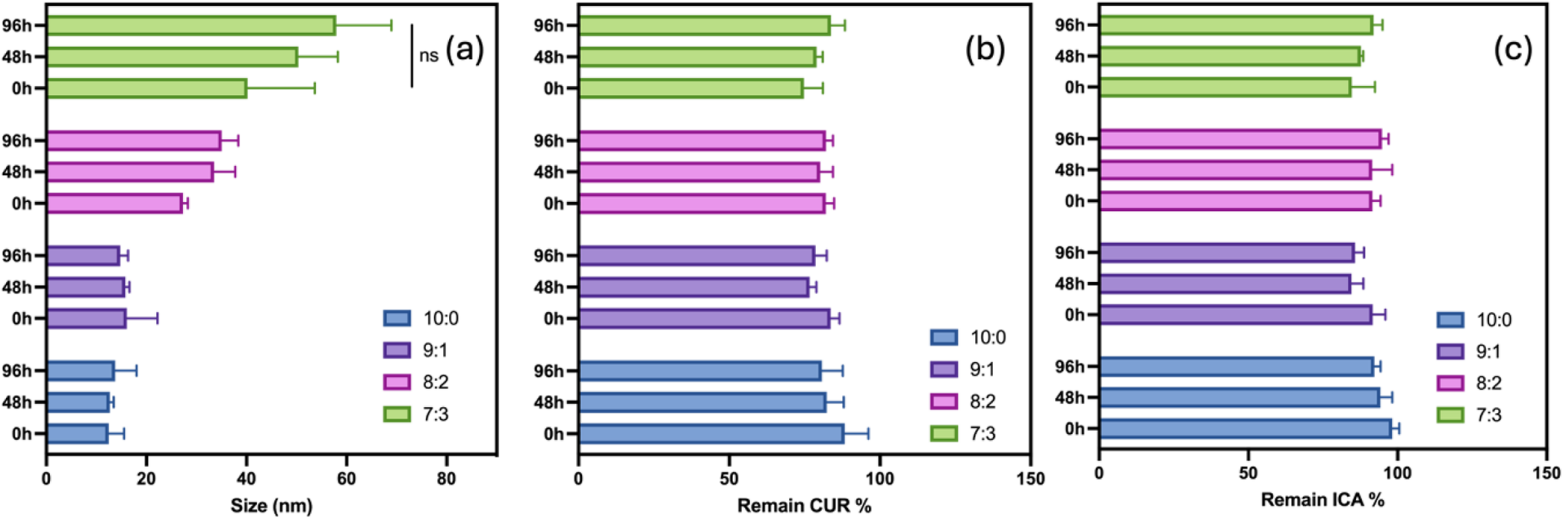
Stability study of ICA + CUR-loaded micelles. (a) Particle size stability of ICA + CUR-loaded TPGS/DPPC micelles at different ratios over 0 h, 48 h, and 96 h. (b) Retention of CUR in the micelles over time. (c) Retention of ICA in the micelles over time.

The stability of particle size and drug encapsulation was assessed over 96 h at 4 °C, and the results are presented in Figure 3. Drug-loaded micelles were stored at 4 °C, and DLS analysis was performed at 0, 48, and 96 h to evaluate particle size stability.

Regardless of the time point, micelles with a TPGS/DPPC ratio of 7:3 (w/w) exhibited the largest particle size, while decreasing DPPC proportions resulted in smaller particles. No significant changes in size were observed over 96 h, confirming the colloidal stability and structural integrity of the formulations. A slight size increase was detected in the 7:3 (w/w) formulation at 96 h, but statistical analysis (*t* test) indicated that this change was not significant.

The drug retention stability of ICA and CUR was further analyzed using HPLC at 0, 48, and 96 h. Approximately 95% of ICA and CUR remained encapsulated in all formulations after 96 h, demonstrating that the micelles-maintained drug retention over the tested period.

### 3.4 Fourier Transform Infrared Spectroscopy (FT-IR)

FT-IR was employed to analyze drug-polymer interactions and assess the stability of encapsulated drugs by comparing the spectra of pure compounds and drug-loaded nanoparticles. FT-IR analysis was conducted to investigate potential chemical interactions among CUR, ICA, lyophilized TPGS/DPPC micelles, and lyophilized CUR + ICA/TPGS/DPPC micelles.

**Figure 4:**
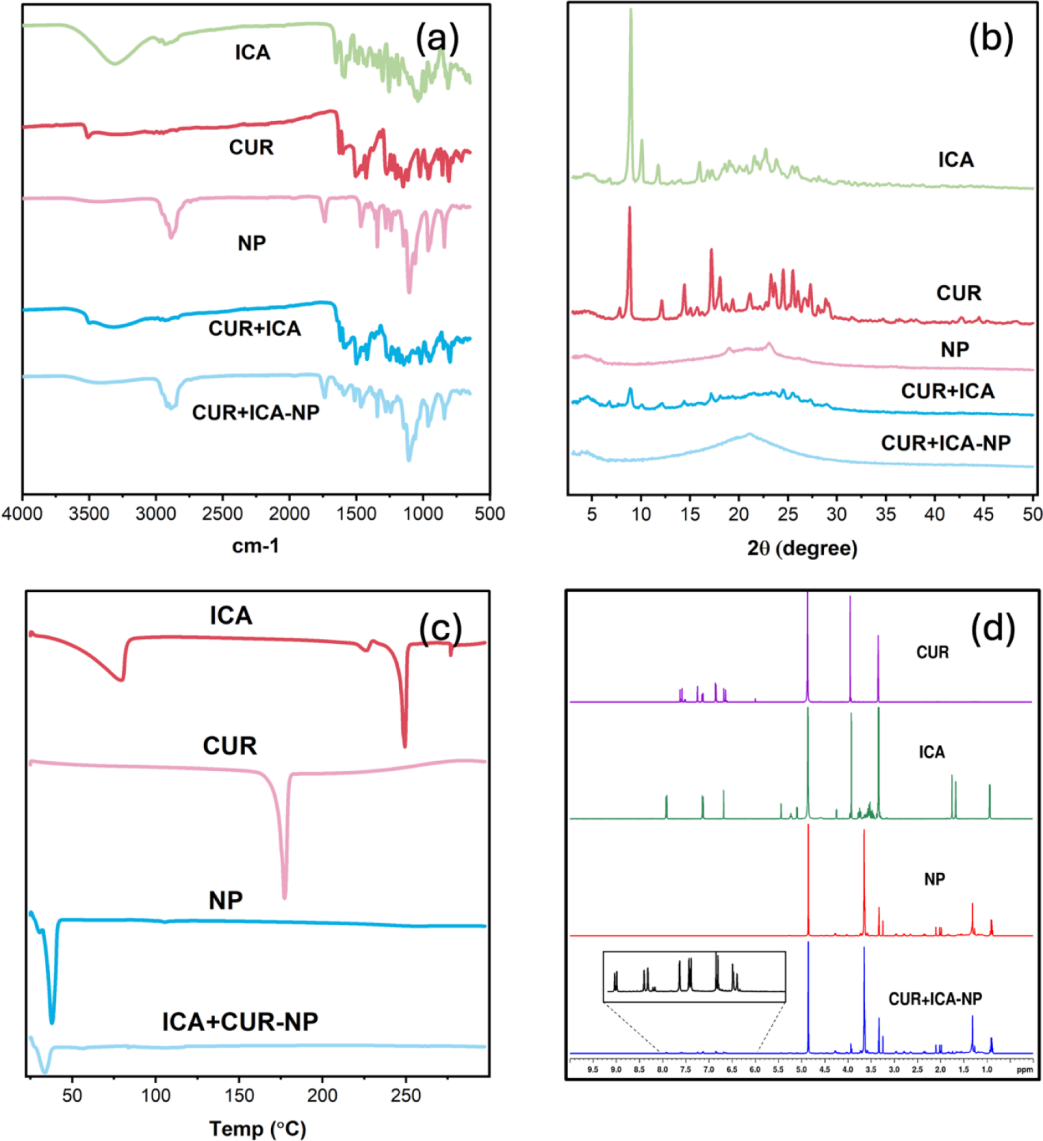
(a) FTIR spectroscopy analysis of CUR, ICA, blank TPGS/DPPC micelles, a physical mixture of CUR + ICA, and CUR + ICA-loaded TPGS/DPPC micelles. (b) XRD patterns of ICA, CUR, DPPC, blank TPGS/DPPC micelles, a physical mixture of ICA and CUR, and CUR + ICA-loaded TPGS/DPPC micelles. (c) DSC thermograms of ICA, CUR, blank TPGS/DPPC micelles, and CUR + ICA-loaded TPGS/DPPC micelles. (d) ^1^H NMR spectra of CUR, ICA, blank TPGS/DPPC micelles, and CUR + ICA-loaded TPGS/DPPC micelles (400 MHz, methanol-d).

As shown in Figure 4a, the FT-IR spectrum of pure ICA exhibited characteristic peaks corresponding to primary alcohol (1073 cm^–1^), aromatic rings (1509 cm^–1^), alkenes (1670 cm^–1^), and phenolic hydroxyl groups (3440 cm^–1^). Similarly, CUR displayed peaks associated with aromatic rings (1278 cm^–1^), olefinic bonds (1428 cm^–1^), benzene rings (1597 cm^–1^), aromatic moieties (1628 cm^–1^), and phenolic hydroxyl groups (3508 cm^–1^).

These characteristic peaks were absent in the spectrum of TPGS/DPPC micelles without the drugs. However, they reappeared in the spectra of the physical mixture of CUR and ICA, as well as in the CUR + ICA-loaded TPGS/DPPC micelles, confirming successful drug encapsulation.

Importantly, no new peaks were observed in the FT-IR spectra of the CUR + ICA-loaded micelles, suggesting that encapsulation occurred through physical entrapment rather than chemical interaction.^49^ This indicates that CUR and ICA were incorporated into the micellar system without altering their molecular structures.

### 3.5 X-Ray Diffraction (XRD) Analysis

XRD analysis was performed to determine the physical state of CUR and ICA within the micelles. Successful drug encapsulation would result in a diffraction pattern distinct from that of the individual drug components.

As shown in Figure 4b, ICA exhibited characteristic crystalline peaks at 2θ values of 5°, 8°, and 9°, while CUR displayed peaks at 2θ values of 8°, 13°, 15°, and 18°, confirming their crystalline nature. These peaks were also present in the physical mixture of CUR, ICA, TPGS, and DPPC, indicating that the drugs remained in their crystalline state when physically mixed.

However, in the CUR + ICA-loaded TPGS/DPPC micelles, these crystalline peaks were absent, and the diffraction pattern resembled that of blank TPGS/DPPC micelles, with no sharp crystalline peaks observed. This suggests that CUR and ICA were transformed into an amorphous state upon encapsulation.^50^

The conversion from crystalline to amorphous form is beneficial for enhancing drug solubility, which may improve dissolution properties and facilitate more efficient drug delivery.^51,52^

### 3.6 Differential Scanning Calorimetry (DSC)

DSC was performed to evaluate the thermal behavior of CUR and ICA after encapsulation within the micelles.^53^Figure 4c presents the DSC thermograms of CUR, ICA, blank TPGS/DPPC micelles, and CUR + ICA-loaded micelles.

Pure CUR and ICA exhibited sharp endothermic peaks at approximately 177 and 248 °C, respectively, corresponding to their melting points, indicating their crystalline nature. However, in the CUR + ICA-loaded micelles, these peaks were absent, and the thermograms resembled those of blank TPGS/DPPC micelles.

The disappearance of these melting peaks suggests that CUR and ICA transitioned from a crystalline to an amorphous or molecularly dispersed state within the micelles. This transformation may enhance drug solubility and dissolution properties, supporting the potential for improved drug delivery.

### 3.7 Nuclear Magnetic Resonance (NMR) Spectroscopy

NMR spectroscopy is widely used for structural determination and provides higher specificity and sensitivity compared to FT-IR.^54^ To confirm that the preparation process did not alter the chemical structures of CUR and ICA, ^1^H NMR analysis was performed on pure drugs and micellar formulations.

As shown in Figure 4d, ^1^H NMR spectra of CUR, ICA, blank TPGS/DPPC (9:1, w/w) micelles, and CUR + ICA-loaded TPGS/DPPC (9:1, w/w) micelles were obtained. Both CUR and ICA exhibited aromatic proton signals between δ_H 6.00–8.00 ppm, while micelles, composed primarily of aliphatic chains from TPGS and DPPC, showed proton signals below δ_H 4.0 ppm, leaving the aromatic region unoccupied.

In the CUR + ICA-loaded micelles, characteristic signals of CUR and ICA remained unchanged, with no shifts, loss of peaks, or appearance of new signals. These findings indicate no chemical interactions between CUR, ICA, and the excipients, confirming that CUR and ICA remained structurally intact without any chemical modification or degradation during micelle preparation.

### 3.8 *In Vitro* Aerosol Performance

The lung deposition characteristics and aerodynamic particle size distribution of CUR and ICA-loaded TPGS/DPPC micelles were evaluated using NGI to assess their pulmonary delivery potential.

For effective nebulization, aerosolized particles must traverse various regions of the respiratory tract. Studies indicate that a flow rate of 15 L/min facilitates nanoparticle deposition in the mouthpiece adapter, throat, and NGI stages 1–3, suggesting initial retention in the upper respiratory tract.^55^ Particles collected in stages 4 and 5 may reach the central airways, while those deposited in stages 6 and 7 can penetrate the peripheral lung regions.

**Figure 5:**
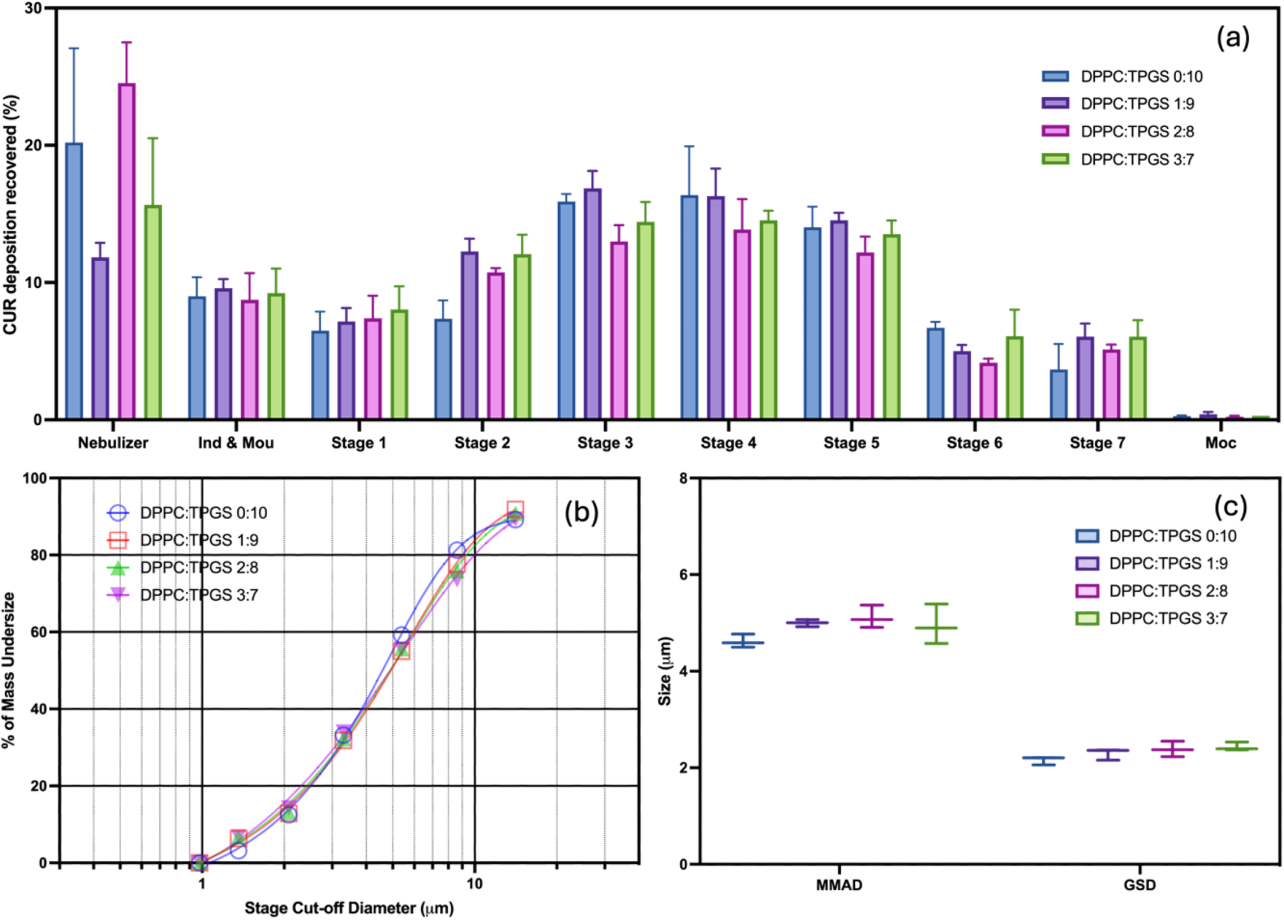
NGI experimental results analyzing the airway deposition of CUR and ICA-loaded TPGS/DPPC micelles with varying TPGS/DPPC ratios. (a) Airway deposition profile, (b) cumulative particle size distribution measured by NGI, and (c) MMAD and GSD values calculated from the cumulative particle size distribution. Data are presented as mean ± SD, *n* = 3.

As shown in Figure 5a, the micelles exhibited favorable aerosolization properties, with more than 50% of the drug recovered in stages 3–7, indicating potential pulmonary deposition. The 9:1 (w/w) TPGS/DPPC formulation demonstrated the highest deposition efficiency, with approximately 60% of the drug collected in these stages.

The aerodynamic performance of the micelles was further assessed using emitted fraction (EF), fine particle fraction (FPF), mass median aerodynamic diameter (MMAD), and geometric standard deviation (GSD).^56^ The micelles exhibited high emitted fractions, with 90–95% of the drug released from the nebulizer, demonstrating efficient aerosolization. The fine particle fraction (FPF) values ranged from 64% to 71%, indicating that a significant proportion of the aerosolized droplets were <5.39 μm, supporting efficient lung deposition.

The MMAD and GSD values were derived from the particle size distribution curves (Figure 5b). As shown in Figure 5c, MMAD values (∼5 μm) fall within the recommended aerodynamic range for inhalable formulations, facilitating deep lung deposition and therapeutic efficacy.^57^ The GSD values (∼2 μm) align with previously reported nebulizable nanoparticles, indicating a consistent particle size distribution essential for reproducible therapeutic delivery.^58,59^

The high FPF, optimized MMAD, and uniform GSD confirm that CUR and ICA-loaded micelles exhibit aerodynamic properties suitable for deep lung deposition, making them promising candidates for pulmonary drug delivery. The micelle formulations also improved the aqueous dispersibility of CUR and ICA, facilitating efficient nebulization and pulmonary administration, which may enhance their therapeutic potential for lung cancer treatment.

### 3.9 DPPH Assay

**Figure 6:**
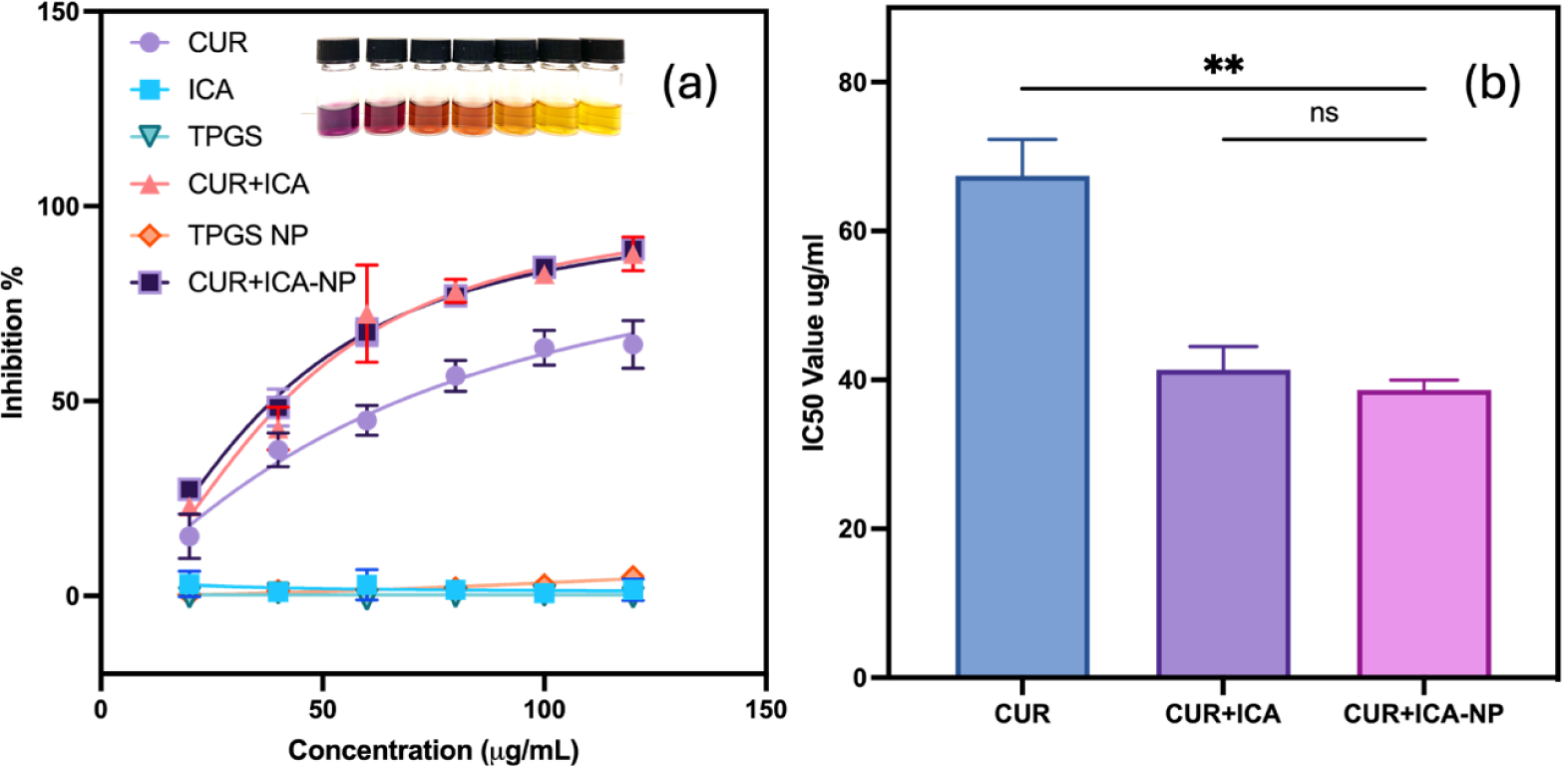
Determination of DPPH radical scavenging activity of CUR, ICA, TPGS, CUR + ICA physical mixture, TPGS micelles, and CUR + ICA loaded TPGS/DPPC (9:1, w/w) micelles (a) and DPPH IC_50_ of CUR, CUR + ICA mixture, and CUR + ICA loaded TPGS/DPPC (9:1, w/w) micelles (b), *n* = 3.

The free radical scavenging activity of CUR, ICA, a CUR + ICA mixture, and CUR + ICA-loaded TPGS/DPPC micelles was evaluated using the DPPH assay (Figure 6a). The CUR+ICA mixture and CUR + ICA-loaded micelles exhibited similar scavenging activity, with 85% and 84% inhibition at 100 μg/mL, respectively. In contrast, ICA alone showed negligible antioxidant activity (∼1%).

The IC_50_ values for CUR, the CUR + ICA mixture, and CUR+ICA-loaded micelles were 67 ± 5 μg/mL, 41 ± 3 μg/mL, and 39 ± 1 μg/mL, respectively (Figure 6b). The comparable IC_50_ values of the CUR + ICA mixture and CUR + ICA-loaded micelles suggest that encapsulation preserves the functional integrity of CUR without altering its intrinsic properties. The radical-scavenging activity observed in CUR + ICA-loaded micelles is primarily attributed to CUR, which donates hydrogen atoms from its phenolic hydroxyl groups to neutralize free radicals, including DPPH radicals.^60^

### 3.10 A549 Cell Viability

**Figure 7:**
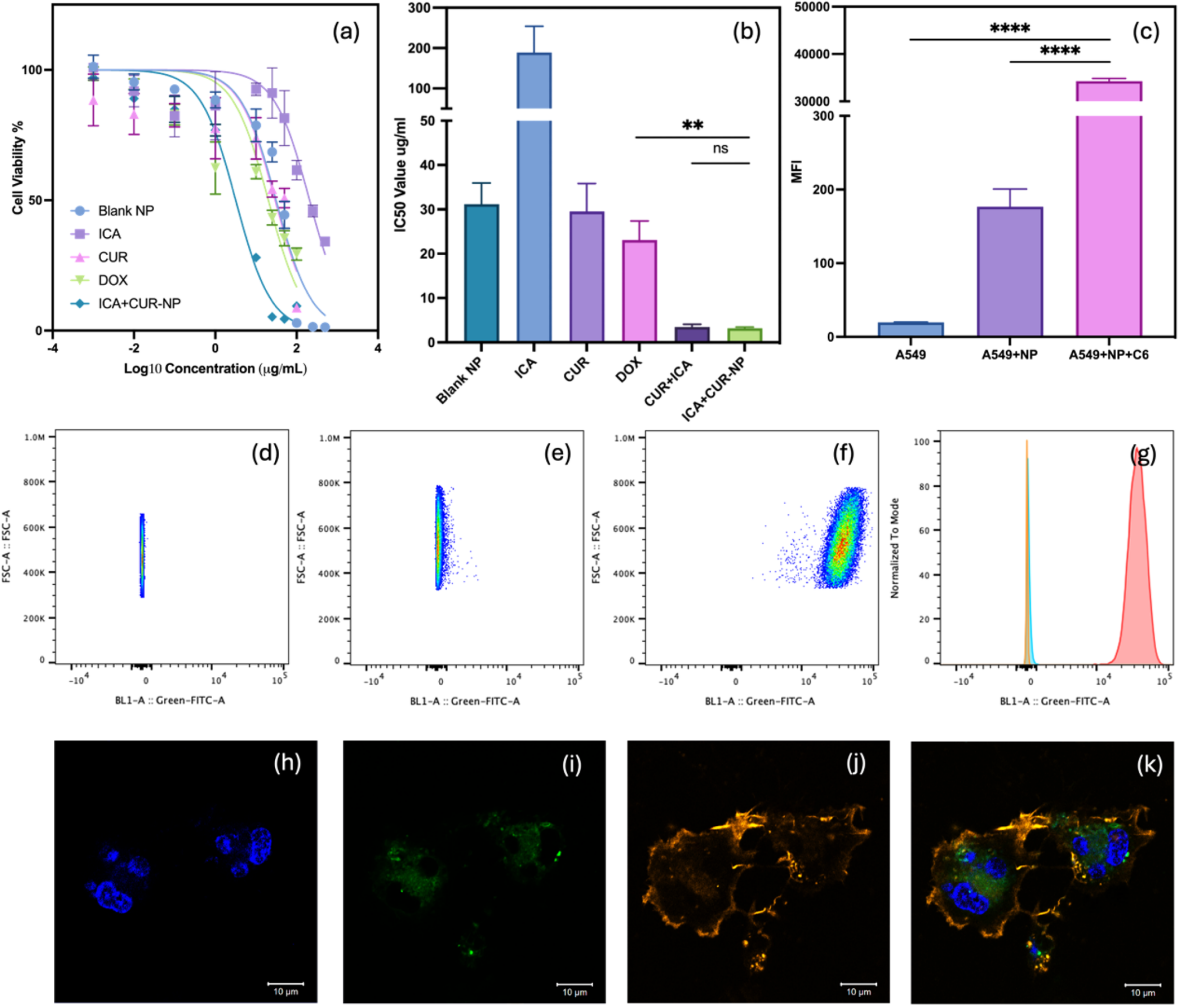
In vitro cell viability assay and cellular uptake analysis. (a) Cell viability dose–response curves of blank TPGS/DPPC micelles, ICA, CUR, DOX, and CUR + ICA-loaded micelles. (b) IC50 values of blank TPGS/DPPC micelles, ICA, CUR, DOX, CUR/ICA physical mixture, and CUR + ICA-loaded micelles in A549 cells. (c) MFI of untreated A549 cells, A549 cells treated with blank TPGS/DPPC micelles, and A549 cells treated with coumarin-6-loaded TPGS/DPPC micelles, measured by flow cytometry. (d–f) Dot plots of A549 cells, blank TPGS/DPPC micelle-treated cells, and coumarin-6-loaded TPGS/DPPC micelle-treated cells, color-coded to show event density in flow cytometry. (g) Histogram comparison of fluorescence intensity among A549 cells, blank TPGS/DPPC micelle-treated cells, and coumarin-6-loaded TPGS/DPPC micelle-treated cells in flow cytometry. (h–k) Confocal microscopy images showing cellular uptake: (h) Cell nuclei stained with Hoechst dye (scale bar = 10 μm), (i) coumarin-6-loaded TPGS/DPPC micelles (scale bar = 10 μm), (j) cell membranes stained with Cell Mask Orange Actin Tracking Stain (scale bar = 10 μm), and (k) merged image of A549 cells (scale bar = 10 μm). Data represent mean ± SD, *n* = 3.

An MTT assay was conducted to evaluate the cytotoxic effects of various formulations on A549 lung cancer cells. Cells were treated with blank TPGS/DPPC (9:1, w/w) micelles, ICA, CUR, doxorubicin (DOX), and CUR + ICA-loaded TPGS/DPPC (9:1, w/w) micelles at concentrations ranging from 0.001 μg/mL to 500 μg/mL for 48 h at 37 °C. Figure 7a depicts A549 cell viability following treatment with these formulations.

The results revealed a concentration-dependent decrease in cell viability across all treatments. The CUR + ICA-loaded micelles exhibited the most pronounced cytotoxicity, reducing cell viability at lower concentrations compared to other formulations. ICA alone showed minimal cytotoxic effects, even at higher concentrations, whereas blank TPGS/DPPC micelles, CUR, and DOX induced moderate reductions in cell viability.

The half-maximal inhibitory concentration (IC_50_) values, presented in Figure 7b, further support these findings. ICA exhibited the highest IC_50_ value (∼200 μg/mL), indicating low cytotoxicity. The IC_50_ values for blank TPGS/DPPC micelles, CUR, and DOX were approximately 30 μg/mL, whereas CUR + ICA-loaded micelles demonstrated the lowest IC_50_ value of 3 ± 0 μg/mL, significantly lower than DOX.

The cytotoxicity of blank micelles may be associated with Vitamin E TPGS, which has been reported to disrupt membrane integrity, induce oxidative stress, and interfere with mitochondrial function.^40^ These characteristics highlight the potential utility of TPGS-based carriers in anticancer drug delivery, not only enhancing drug solubility but also contributing to tumor-selective cytotoxicity.

These findings suggest that CUR + ICA-loaded micelles exhibit enhanced anticancer activity, likely due to the synergistic effects of CUR and ICA. The significantly lower IC_50_ value indicates greater potency, which may translate to improved therapeutic efficacy in lung cancer treatment.

### 3.11 Qualitative and Quantitative Cellular Uptake

Figure 7h–k illustrates the qualitative cellular uptake of fluorescently labeled micelles, as observed via confocal microscopy. A549 lung cancer cells were treated with coumarin-6-loaded TPGS/DPPC (9:1, w/w) micelles. Hoechst dye (blue) stained the nuclei, while CellMask Orange Actin Tracking Stain marked the cell membranes. The green fluorescence of coumarin-6-loaded micelles localized around the nuclei, suggesting effective internalization of micelles by A549 cells, facilitated by the TPGS/DPPC formulation.

Figure 7d–g presents the quantitative assessment of micelle uptake using flow cytometry. The mean fluorescence intensity (MFI) (Figure 7) was significantly higher in coumarin-6-loaded micelle-treated cells compared to the blank micelle or control groups. These flow cytometry findings align with confocal microscopy results, confirming efficient cellular uptake of TPGS/DPPC micelles.

As previously noted, poorly soluble drugs like CUR and ICA exhibit limited membrane permeability, complicating their direct cellular uptake. Micelle encapsulation enhances drug solubility, promotes efficient endocytosis, and improves intracellular retention, thereby potentially optimizing therapeutic efficacy.^61^

## 4 Conclusion

This study demonstrates the potential of CUR and ICA-loaded TPGS/DPPC micelles as a pulmonary drug delivery system for NSCLC. The combination of TPGS and DPPC, a pulmonary surfactant component, enabled effective inhalation-based delivery of these hydrophobic drugs, while enhancing their solubility, stability, and cellular uptake. The optimized 9:1 TPGS/DPPC formulation exhibited favorable particle size, high encapsulation efficiency, and stability, supporting its suitability for further investigation.

In vitro cytotoxicity studies confirmed that CUR and ICA-loaded micelles significantly reduced A549 lung cancer cell viability, demonstrating greater cytotoxicity than doxorubicin. Aerosol performance evaluations revealed high fine particle and emitted fractions, supporting their potential for deep lung deposition and localized drug delivery.

The CUR and ICA-loaded micellar system offers a promising noninvasive strategy for NSCLC therapy, addressing key challenges such as multidrug resistance and poor aqueous solubility. The formulation’s optimized physicochemical properties and favorable aerosol performance support its feasibility for pulmonary administration. Further preclinical and clinical studies are necessary to validate its therapeutic efficacy, safety, and translational potential for NSCLC treatment.

## Data Availability

The data underlying this study are available in the published article and its Supporting Information.

## Supporting Information Available

HPLC chromatograms of ICA and CUR combination; calibration curve of ICA; calibration curve of CUR (PDF)
