# Supplementary material for: Inhalable TPGS/DPPC Micelles Coloaded with Curcumin and Icariin for Targeted Lung Cancer Therapy
Source: ACS Omega. 2025 Apr 11;10(15):15400–11. doi: 10.1021/acsomega.5c00008 (PMC12019740; doi:10.1021/acsomega.5c00008)
Supplement: Supplementary file 1 — ao5c00008_si_001.pdf [file ao5c00008_si_001.pdf]

Inhalable TPGS/DPPC Micelles Co-Loaded with Curcumin and Icariin for Targeted Lung Cancer Therapy

Chengwei Jiang, Rongjun Bai, Satyanarayana Somavarapu*

1 Department of Pharmaceutics, School of Pharmacy, University College London, 29-39 Brunswick Square, London WC1N 1AX, UK

* s.somavarapu@ucl.ac.uk

Supporting Information

1. HPLC chromatograms of ICA and CUR combination 2

2. Calibration curve of ICA 2

3. Calibration curve of CUR 3

1. HPLC chromatograms of ICA and CUR combination

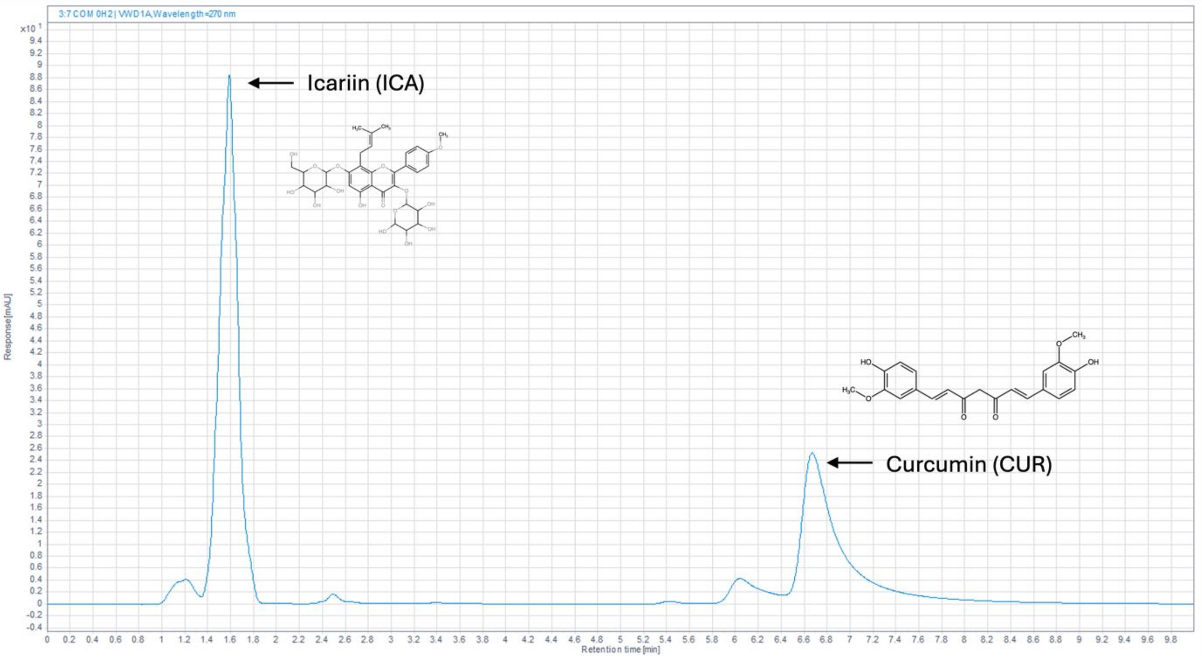

Figure S1. *HPLC chromatograms of ICA and CUR combination.*

2. Calibration curve of ICA

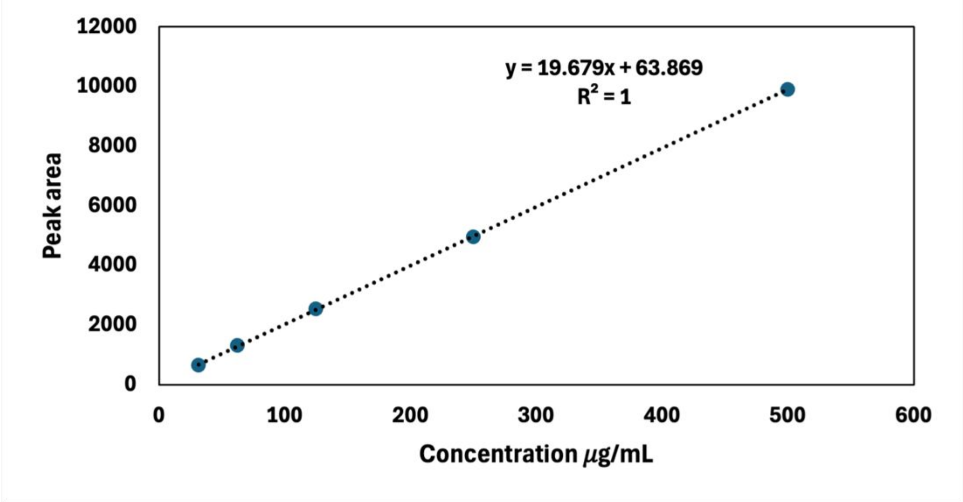

Figure S2. *HPLC calibration curve of ICA concentration.*

3. Calibration curve of CUR

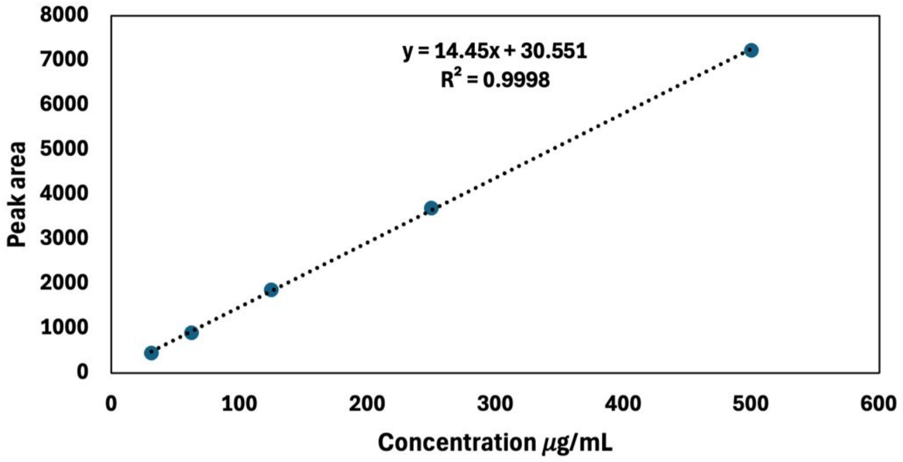

Figure S3. HPLC calibration curve of CUR concentration
